# Supplementary material for: Understanding perceptions of schistosomiasis and its control among highly endemic lakeshore communities in Mayuge, Uganda
Source: PLoS Negl Trop Dis. 2023 Jan 19;17(1):e0010687. doi: 10.1371/journal.pntd.0010687 (PMC9888691; doi:10.1371/journal.pntd.0010687)
Supplement: S1 File — (DOCX) [file pntd.0010687.s001.docx]

**S1 File – Raw data analysed in** “**Understanding perceptions of schistosomiasis and its control among highly endemic lakeshore communities in Mayuge, Uganda.”**

Lazaaro Mujumbusi^1,2*^, Edith Nalwadda^1^, Agnes Ssali^1^, Lucy Pickering^2^, Janet Seeley^1,3^, Keila Meginnis^2,4^ and Poppy H. L. Lamberton^4,5*^.

1. Medical Research Council/Uganda Virus Research Institute & London School of Hygiene and Tropical Medicine, Uganda Research Unit, Entebbe, Uganda
2. Institute of Health and Wellbeing, University of Glasgow, Glasgow, UK
3. London School of Hygiene and Tropical Medicine, London, UK
4. School of Biodiversity, One Health & Veterinary Medicine, University of Glasgow, Glasgow, UK
5. Wellcome Centre for Integrative Parasitology, University of Glasgow, Glasgow, UK.

[Lazaaro.Mujumbusi@mrcuganda.org](mailto:Lazaaro.Mujumbusi@mrcuganda.org) (LM), [Poppy.Lamberton@glasgow.ac.uk](mailto:Poppy.Lamberton@glasgow.ac.uk) (PHLL)

**List of Abbreviations**

**LM:** Lazaaro Mujumbusi (Interviewer)

**EN:** Edith Nalwadda (Interviewer)

**FGD:** Focus Group Discussion

**IDI:** In-depth Interview

**OB:** Observation

**PO:** Participant observation

**TW:** Transect Walk

**VHT:** Village Health Team member

**Perceptions about symptoms of bilharzia**

**2018.01.25 BWONDHA FGD LM**

I: How can you tell that someone has bilharzia?

P2: You can easily detect because a person with bilharzia doesn’t look like other people, the eyes become white, sunken, the stomach swells and he looks bad.

P5: We can tell after doing a check-up because someone can have a swollen stomach when he is not sick.

P4: Personally whoever I see with a swollen stomach I know he has bilharzia.

**2017.11.10 Musubi FGD LM**

I: How can you tell that an adult has bilharzia?

P9: Such a person, the stomach swells slowly up to when it becomes big.

P5: He also turns yellowish.

P2: Even if that person has not gone for check-up, for us we know that whoever has a swollen stomach has bilharzia as long as he is not bewitched.

**2017. 12.18 MUSUBI IDI LM**

I: After telling you that it is ekidada, what kind of treatment did you receive?

R: Local herbs.

I: Where did you get it from?

R: I got it outside Musubi, I went to Bugweri and Busiki.

I: Who did you go to?

R: Witch doctors.

I: What kind of treatment did they give you?

R: Herbs.

I: Where they for drinking or?

R: For drinking. One gave me herbs to drink in porridge or millet and others gave me plane herbs for drinking.

I: Have they worked?

R: All the herbs have not worked, it seems the person who bewitched me, also added charms of not getting healed, that is why you see up to now I look like this as you are seeing (swollen body and stomach). The witch doctor I went to in Bukhooli made me to defecate a toad but the stomach is still swelling.

**2018.03.23 Bwondha IDI LM**

I: Where did you learn about bilharzia from?

R: I got to know about bilharzia through the bilharzia workshops I have attended for years and years by health workers who are in charge of bilharzia, that is when i got to know it is true there are parasites that make the stomach swell but not ekidada that is bewitched.

**2018.02.08 Bwondha FGD EN**

I: What do you think could be the cause?

R.1: I also do not know.

R.7: Since she swims a lot in the lake, she could be having bilharzia

R.1: But there are men who have big stomach but do claim that they have money

R.7: Yet they are suffering from bilharzia

R.1: When they have bilharzia

R.8: But it depends; am trying to differ from R.1 because there are stomachs which do not reflect a wealthy person

R.4: So you are the only one who can differentiate stomachs of wealth people!

(Laughter)

**2018.02.08 Bwondha FGD LM**

P3: Me I know bilharzia it is something that turns one into another thing, and someone will be labelled rich because of a big berry yet it is not richness but illness. Those parasites are found in lakes like in a snail.

**2017.10.12 Bugoto FGD LM**

P2: They tell us that bilharzia is a parasite just the way HIV is a virus only that for bilharzia you catch it from water and am so eager to have a look at that parasite and see how it looks like. I have my brother XX, his feet swell and people say that he acquired that parasite that made the feet to swell from the lake, and they say it is that bilharzia so I would like to know what bilharzia is? And I would like to look at the parasite that causes it and know how it looks like so that if am in the lake and I happen to see that parasite I can avoid it.

**2017.09.25. BUGOTO IDI LM**

I: How about adults?

R: For the adult to get to know that he has bilharzia…., you get to know from the stomach, the stomach swells, sometimes the limbs swell. Some people even say they have HIV but when they have bilharzia.

I: Why do they say that they have HIV?

R: They become slim when it is only the stomach that is big on them.

**2018.01.25 Bwondha FGD LM**

I: How can you tell that someone has bilharzia?

P2: You can easily detect because a person with bilharzia doesn’t look like other people, the eyes become white, sunken, the stomach swells and he looks bad.

P5: We can tell after doing a check-up because someone can have a swollen stomach when he is not sick.**2017.09.12 Bugoto FGD LM**

I: How can you tell when adults have bilharzia?

R.5: Some present with signs like anaemia, becoming pale and general body weakness

**2017.11.10 Musubi FGD EN**

I: How about in children?

P6: For children who play from the lake and also swim it is easy to know because they always complain of abdominal pain, general malaise and diarrhea.

**Perceptions about the treatment of schistosomiasis**

**2017.11.04. Musubi FGD LM**

P9: Me I was an original fisher man and am still a fisher man, I remember my first time to swallow those tablets, I didn’t even sleep at home, I looked as if I was dead, they found me in the maize plantation and took me back home, I was not feeling well, by then I had a swollen stomach as If am rich yet am poor but after two years when I took the tablets again there was some change because I didn’t feel like the first time I took those tablets, of recent I swallowed but I didn’t get any side effect, so bilharzia can be treated.

**2017.10.05 Bugoto PO LM**

He asked me who am I, then the bar attendant I talked to during mobilization told him am a health worker conducting research on bilharzia, this talkative man said while he was in Bwondha he took bilharzia drugs but he got diarrhea and didn’t go to work. That he just put the mat near the toilet so that he could run to the toilet at any time, that he became weak after and had to go for further treatment, he said ever since that time he will never take bilharzia drugs again. He continued and told me he was telling me the truth about the drugs, that people hide because they fear side effects.

**2017.09.25. BUGOTO IDI- LM**

I: What else affects MDA?

R: It is what I have told you already, they fear to swallow those tablets that they will make them get diarrhoea. Some people say they reduce sexual man power, some people say that those tablets are like a method of family planning, that when you swallow them a lot you don’t produce.

**2018.02.08 Bwondha FGD LM**

P9: Me I think the tablets they give to people are not important, there is a child who stays in XX, they have given him those tablets but the stomach failed to reduce, it is still swollen, they have given him tablets several times, whenever they come they give him but the tablets failed to respond to the illness. So I think those tablets don’t work.

**2018.03.23 Bwondha IDI- LM**

I: So what are the barriers to MDA?

R: Apart from ignorance there is also some religion that do not allow such things, in the religion of ngiri nkalu people don’t swallow tablets, they are not injected, they don’t educate their children, when we are immunizing for polio we just fight with them and take them to police.

**2017. 12.18 MUSUBI IDI- LM**

I: Why did you decide to go to for tablets?

R: I thought I was suffering from bilharzia because I had spent long time fishing, I thought I had acquired bilharzia because they say that ekidada caught through water contact makes the stomach swell, I went and she gave me tablets but they did not respond. We went to XX health centre IV, there is an in law of ours who works in Mayuge, you might be knowing that man because he was working in XX hospital, I went and he checked me, he told me that I had a heart problem and a liver problem, he then told me to get 40,000UGX to get me tablets. We gave him and he got us tablets, I swallowed them but still they also didn’t respond to my illness, then people told me I was suffering from ekidada that needed local herbs to get healed.

**2018.02.16 Bwondha IDI- LM**

I: Is there another reason as to why people don’t swallow?

R: The VHTs move door to door giving out tablet but some people refuse to swallow, they tell them, for me I don’t have bilharzia as if they have ever tested.

**Perceptions about how schistosomiasis is contracted**

**2018.02.12 Bwondha IDI- LM**

I: Let us now categorize, how do adults catch bilharzia?

R: Adults catch bilharzia through drinking unboiled water, and even if you boil the water and use a wet mag to drink boiled water you still catch bilharzia. That water mixes with the unboiled water.

**2018.03.10 Bugoto FGD LM**

I: How do children catch bilharzia?

P1: Through swimming in the lake.

P8: They play from the lake.

P3: They swim from the lake and start swallowing that water.

I: What kind of water do you call dirty?

P5: Children swallow that water while swimming.

**2017.10.10 BUGOTO IDI- EN**

R: Someone that goes to the lake might contract bilharzia from the water, and also one that normally gets in contact with dirty water for example those that are in rice farming in the wetlands, also if they go into the lake, you might contract bilharzia.

**2018.03.14 BWONDHA FGD LM**

I mobilized these participants with the help of a male VHT, we started with four men who we found at the lake two of them dis entangling nets and other two were just seated at the lake shore conversing. The VHT called them together and introduced me to them, I also introduced myself and explained to them the study. They all accepted to participate. One of them told me that bilharzia is rampant because people don’t swallow drugs. I told them the meeting time was at 2:00pm and asked them whether they will be free by then. They told me at that time they will be done with fishing and assured me they will attend. I thanked them and left, we continued to Mr XX who we found at a sports betting place. The VHT called him a side, I introduced myself and explained to him the study, he told me he will participate, “he further told me that bilharzia has affected people of Bwondha, that some people take long to know that they are suffering from bilharzia because it can take even 10 years without someone presenting any sign of bilharzia, that to him they cannot avoid catching bilharzia because it is found in the lake where they work from, and that it is where they get water from”.

**2018.02.16 Bwondha IDI- LM**

I: How else do people catch bilharzia?

R: They also catch through defecating everywhere.

I: How?

R: You may defecate where they have already defecated and the parasites around may enter you.

**2018.03.10 Bugoto FGD LM**

**I: what were you saying?**

P3: We children catch bilharzia because we don’t put on bidco (bidco are plastic sandals) and when it rains we step-in earth worms, in case anything pierces the feet then bilharzia enters.

P8: Children who dig from swamps also catch from bilharzia.

P5: Children who step where they have defecated also catch bilharzia.

**2018.03.13 BWONDHA IDI- EN**

R: If you walk barefooted in dirty places. Like now, this is a rainy season but some body may decide to walk bare feet yet the place is dirty and it has cow dung, so this exposes one to bilharzia.

**Perceptions about schistosomiasis onwards transmission and responsibility of transmission**

**2017.09.14 BUGOTO IDI- EN**

I: Who are those ones that cause bilharzia?

R: They are the fishermen.

I: Is it the fisher men that cause bilharzia?

R: It is because they are the ones that usually misuse the lake by having to urinate in it, once nature calls they can’t urinate anywhere else but rather in the lake.

I: Urinating? Don’t they defecate in it?

R: They also defecate in there, they will do like this (Demonstrated how they bend on the boat) at the lake and defecate in the lake.

I: You mentioned that there are people who defecate on the sides but are there people who defecate in the lake?

**2017.09.25 BUGOTO IDI - EN**

R: With majority of fishermen, the lake is their toilet. Even when they feel like defecating while here at the landing site; they will hold up the faeces until they get back to lake since they have no option. When he goes to the lake, he can just open his trousers, bend a little with the support of the boat edges and he defecates. The lake is a toilet for fishermen. The reason why they do not want to defecate in the toilets is because they do not have them, all they do is defecate in the lake

**2017.11.04. MUSUBI FGD LM**

I: What makes home toilets dirty?

P5: It is family members who make it dirty.

P4: On that issue of the toilet, you have asked why they leave home toilets and poo at the lake but the issue is, people who work at the lake are mature and they go at the lake for work, if they feel like defecating they can’t go back home when at the lake there is a natural toilet.

I: What is that natural toilet?

(All laughs) p9: The lake.

**2017.11.01 Musubi Ob LM**

This youths who asked me about the child then told me that “For us the problem that we have is that we don’t have toilets, we don’t even have a public toilet, those who have rental houses built them but without toilets so we rent houses without toilets, am telling you, if you stand here you can’t take 10 minutes without seeing any one going that side to defecate (he pointed at the bush at the lake shore) I will not lie to you that it is other people who go there, even me I defecate there, so health workers you should be strict on people who build houses without toilets so that they are penalized, you should take the initiative to visit homes and inspect those without toilets”. I told them we shall go to all those places of open defecation, then they left and we also moved up towards the area of open defecation. This place was smelly because it was near the very place with rubbish near the lake and where people defecate.

**2018.02.08 FGD LM Bwondha**

P: We cannot avoid defecating in the lake because we are always at the lake the whole day. It is unavoidable

**2017.10.11. Bugoto IDI- LM**

R: For me by the time I started fishing, we even used to buy faeces to use in trapping fish, after putting the fishing net you go where the wind is coming from and defecate there so that the faeces flows towards the net, when the faces join the fishing net the fish that were eating the faeces also would like to continue eating and they enter the net. At one point we even bought faeces. We could tell people I need today, then he would defecate on a banana leaf, then covers it well and we go with it in the lake, after putting the fishing net you throw the faeces around and then we trap a lot of fish.

**2017.10.11. Bugoto IDI- LM**

I: You told me your toilets are of three kinds, so where do different people defecate?

R: Majority people especially the XX tribe don’t have latrine, they don’t go to toilets apart from the men, women from the XX tribe if they defecate in a latrine they believe they are throwing their babies in the toilet, if a XX woman goes to the toilet it can make her be slapped.

I: Then where do XX women defecate?

R: They defecate in the bush, if they are at the lake they defecate in the papyrus.

**2017. 09. 14 Bugoto IDI- LM**

R: A person with bilharzia can tell a friend not to eat on the potato he ate on, they should tell friends, go and have yours, don’t drink on my water, don’t drink on mine. you don’t share anything with a person with bilharzia.

**Perceptions about how people can prevent infection and onward transmission**

**2018.02.08 FGD LM Bwondha**

I: Is there anything you do to reduce the risk of catching bilharzia?

P5: For me at this landing site there is no way we can prevent bilharzia because you may tell me not to step in water but I have to fetch water from the lake where the parasites are. You may tell us not to do open defecation but when I don’t have a latrine so I will have to defecate on sand at the lake shore line so it needs government efforts to put in place what would help us reduce the risk.

**2017.11.01 Musubi Ob LM**

So they asked me where are bilharzia parasites born from, I told them it is produced with in the snails, I told them when people defecate in the lake and faeces meet the snails then they hatch the parasites from the snails, I told them in a day one snail can produce over 2000 parasites, they were all amused and said only a day!!!, their faces looked as if they are worried when they heard of this, so then one of them told me “if it like that then we cannot avoid catching bilharzia because for us we take that water and we may catch bilharzia, we work from the lake and we cannot avoid the lake because that’s where we work from, get water for drinking, bathing and washing and sometime we bath from the lake” one continued that they can only avoid bilharzia if they get other jobs other than fishing but if they have no other jobs they will not avoid the lake because that’s the alternative employment that they have and they can’t avoid stepping in water and that when they are sailing lake water sprinkles on them.

**2017.11.10 Musubi FGD- LM**

I: One of you talked of leaving water in the sun, do other people do so?

P5: Those are very few people.

P6: You rarely find them.

P8: Our fore relatives lied to us that when you leave it in sun you have given that water to demons.

P2: We fear to leave water for bathing in the sun, because we were told that when you leave water in the sun, it is ghosts of your ancestors that bath that water first and for you when you bath it you shower yourself with bad omen. That’s why we fear to leave water for bathing in the sun.

**2017.11.10 MUSUBI FGD LM**

P: I will not lie to you that I can waste time to filter water for bathing, that’s haram (forbidden). I get from the lake, pour in a trough and bathe.

**2017.09.15. Bugoto IDI- LM**

R: We draw from the lake because the drinking water that they bring in the community is just sold, so those without money mainly get from the lake… They sell at 700 shillings. people don’t afford to buy that water, that’s why they get drinking water from the lake

**2018.02.16 Bwondha IDI- LM**

R: For us we don’t fear the parasites, our bodies are already strong. Our bodies got strong long ago.

**2018.03.14 BWONDHA FGD LM**

P: What makes us not boil drinking water is that firewood is expensive, one piece is being sold at 300 shillings or 1000 shillings, so people say they can't waste the firewood or charcoal on boiling water yet they have to cook food.

**2017. 12. 11 MUSUBI IDI LM**

I: How about leaving water in the sun?

R: That is so easy, because the jerry can can be placed in the compound and the sunshine can shine on it.

I: Do you purposely leave it in sun or?

R: The sun just finds the jerry can there, it is not done intentionally that we want to leave water in the sun to warm it, the sun just finds water there.

I: Meaning it is not done intentionally?

R: Now the water is there and jerry cans are in the shade (he pointed at water in the sun), after people will bath and still if the sun finds it there they will bath water warmed by the sun.

**2017.10.13. Bugoto IDI- LM**

I: Are there those who boil this water for washing?

R: That is not done. People think about the time and charcoal wasted in boiling water, they even say, how can I boil water just for a cloth? I can’t boil.

I: You told me people don’t boil water, don’t leave in sun and don’t filter but you have said they told you to do, so I would like to know why they do not practice these things they were told.

R: People don’t have that time to first leave it in sun, someone wants to cook food and she has no time to leave water in the sun first, they say, aah! If am to get infected let me be infected, I will go to the hospital.

**2017. 09. 14 Bugoto IDI- LM**

I: Are there those who leave water in the sun?

R: If you have not been used to something it burdens you, for us we have not been knowing that when you leave water in the sun the parasites die, for us after fetching from the lake you pour in the pot and drink, after fetching from the lake you pour in the trough and bath, after fetching from lake you wash utensils or bath babies. And if I find a child leaving water in the sun I will even cane him because the sun destroys the jerry cans, if I find jerry cans in the sun i will be amused because I know they have to be in the house, if they are from fetching they enter them inside, they only come out when they are going again to fetch water, if you leave in the sun a cow can step on it, If a thief looks each side when there is no one seeing him he can just put the jerry can on his head and walks away as if it is his. So the jerry cans should be inside.

**Some extracts from the observations (OB)**

**2017.09.07 Bugoto Ob LM**

We continued towards the first tap but on the way in the trading center eyes were on us because I think this was our third day in the community. We started from the first tap that is opposite XX. We found XX 38-year-old seated in a kiosk breast feeding her child, other three children were besides her, one was swinging on a rope tied on the kiosk, this woman told him to cut it off because they are destroying her kiosk, we found the tap locked and it was dry looking as if it doesn’t function. We introduced ourselves to her as researchers on bilharzia and requested her to tell us the person who is responsible for the tap, she said she is the one, I requested for permission for her to talk to us and she accepted, I asked her whether the tap works and she said that it has taken one year without working. That the XX removed the meter that it was faulty; I asked her how much was she selling her water and she said 100shs. Inquisitively I asked her since she has not been operating for a year where does she think her customers who used to buy water from, she said that they buy from other taps, others buy from wells at XX’s place in XX, I asked her where XX is and she said it is the next village after Bugoto B, that people fetch water from there and sell to them at 500 or 700 and 1000shs during dry season. She said her previous customers now get water for drinking from the well in XX and water for washing from the lake. She said the tap was for her father and gave her authority to run it. We thanked her for talking to us and then left.

We then went to the second tap at Bugoto lake view primary school; here we found a tap but still also with a padlock.

We continued to the fifth tap and passed via women seated under a tree selling tomatoes and cabbages. It had rained at night and the water trenches had stagnant water and ducks were playing from there. We reached at the tap which is at the XX, it has three outlets and it is non-functioning, it has never worked ever since it was constructed according to our guides. They said it was supposed to work with the opening of the spincon (fish market) but the spincon has never been opened.

After noting that we continued to the sixth tap, we found it with a padlock and there was a metal that had even started rusting

**2017.09.08 Bugoto TW LM**

I moved towards the inland area where I found two women washing clothes, two men were bathing from the lake, here there is a lot of water hythene, six men were preparing their boats to set off for fishing while preparing they were conversing that getting what to eat was hard the previous day, I think they didn’t catch fish, one woman was here washing sandals, two boats set off, six children were swimming, another man was on boat sailing dressed in white shirt, grey trouser and open sandals, two men had also come to fetch water, another man also came to wash clothes, two boats also landed.

While here one young man came with clothes in a trough to wash, when he reached he put the trough down, removed his clothes, remained in pant and started swimming, I think he was to wash after swimming, while leaving three children were playing from the lake, another mature man around 50 years had removed his clothes bathing from the lake, I thought if he saw Edith (Observer) he would hide because in Busoga it is shameful someone to look at you necked especially the opposite sex but instead he stood up to look at us, I was also feeling shy to look at him, when he looked at us I just continued without looking back, he was bathing and other women and girls were also coming to fetch water, I talked to our female guide whether people don’t feel a shamed to be seen bathing, she said people don’t feel a shamed that even if a girl is there fetching water or children are there men just remove their clothes and bath, that they say that the one who looks at them is the one who gets a shamed, while going back I still met women who were coming to fetch water then they started conversing, they were saying that in their area girls at the age of 9 already know about sexual matters and they are sexually active, one of them said “there is no value in marriage, what will I gain if I get married, I have to enjoy life, the other one said that girls should get married at 25 because at that time she has enjoyed everything. I left them still conversing.

**2017.09.26 Bugoto Ob LM**

At 3:30pm I went to the lake to observe the activities. It was so hot, the lake had waves and the water was appearing dirty as if there was running water, when I reached at the lake 32 children, 16 women and 1 man were sun drying silver fish, the children sun drying silver fish would pick snails from the silver fish, they were being rewarded with a mag of silver fish or coin not beyond 500UGX (10 UK pence), the children would sell the silver fish between 200-500UGX according to the two children I interacted with, later these children after picking snails from silver fish they would come straight to the lake to play and some went home, 7 children were bathing, 6 boys and one girl were fetching water from the lake, one younger man was washing a motorcycle, 4 boys were disentangling nets, 7 men were playing Ludo, 4 were buying silver fish and they had come from far areas of Bugoto, two men were making a fishing net, two were making boats and four children playing.

While at the lake, I stood exactly were majority people collect the water from in a strategic position that could enable me to view what is taking place in other parts of the lake, while hear one woman came with a child around two years to fetch water, this woman removed her slippers, left the child at the shore line standing, she entered the lake and collected water, after collecting she washed her feet and also made the child stand in water and bathed her from there, another woman also came to fetch water, she had dressed in a black skirt, white blouse and blue slippers, her face looked as if she was tired, she told one of the four boys who were playing from the lake shore to collect for her water and the boy did so, while this boy was collecting water, the woman was saying she fetched water in the morning but used it for washing utensils when she came back from the garden, she urged this boy to collect water quickly because she was hungry that she wanted to go and prepare food. While this boy was fetching water other two girls were also fetching water, this boy was playing from there and sprinkling water on these two girls, this woman again told her to collect water quickly because she was hungry and she wanted to go and prepare food, the boy gave her the water, I was wondering whether he was her son or not, my question was answered when she was leaving, the boy continued playing and she told him that she will not look for him when the food is ready if he continues playing from the lake, that’s when I realized she was his mother but despite the mother’s words the boy continued playing with other friends at the lake shore.

Among the boys who were bathing four of them started washing their clothes directly from the lake, while washing they were discussing about Ugandan musicians and said some are in illuminati, they kept on mentioning the names of those musicians they think are in illuminati. Children who were picking snails from silverfish after finishing their work at the spincon they went to another place where they were sun drying silverfish from at the health facility and they started picking snails from silverfish. One of the woman who was sun drying silver fish later came to the lake and washed the net from the lake, she put it in the lake, washed using hands and later she was just stepping on it to clean it while it was in water. I also saw one engine boat leaving with two younger men on it.

**2017.09.27 Bugoto Ob LM**

The previous day after having done observations at the lake in the afternoon, this day I wanted to observe it in the morning hours to see activities that are carried out in those hours at 10:08am, by the time I reached at the lake it was calm, it had no waves and the water appeared clean. This time I went at the inland area where people mainly bath, swim wash, and fetch water. I found one-man bathing, five people were collecting water, five were washing clothes, three were washing motorcycles, one washing bicycle, and five were seated just resting.

While I was hear one man was washing clothes directly from the lake, he was washing bed sheets, shirts, trouser and also pants, another man also came at the lake and washed from the lake shore but he was fetching water using the trough, seven clothes (shirts and trousers) were hanged down, I guessed they belonged to the four men who were resting because two of them had no shirts, I think they were waiting for them to dry, the other man who was washing started bathing, one woman also came with clothes and started washing, another younger man came with a motor cycle at the lake, he parked it at the in land, removed his sandals, folded his trouser, parked the vehicle in shallow water of the lake and started washing it, another woman also came and started fetching water from the lake and then left. After the gentleman who was bathing left I also went to where he was bathing from, I found it had stones that they step on while bathing and there was also soap left there on these stones, there was also bathing sponge, I don’t know whether someone forgot it there or someone was about to bath, two gentlemen also came at the lake, stood at the lake for some ten minutes and later left.

As I was leaving I found two men conversing and one tall man around thirty years who had put on a white shirt, black trouser and blue slippers told the other that *“At home we have a bath room but I can spend several months without bathing from home because it inconveniences me, you bath and get dry immediately. But at the lake you can bath many times, when you are getting dry you go back”* as this man was talking another man was urinating near the lake. I moved passed the spincon and to the health facility as I was going to observe the market area.

**2017.10.10 Bugoto Ob LM**

As I was making observations I used to find faeces in between house corridors, home compounds and behind the toilets also. In the interviews participants used to say when it rains everything is washed away to the lake by running water. When it started raining this is what I wanted to observe so when it rained I put on my raincoat, umbrella and gum boots and went straight to the lake. Being that the houses are at a sloppy area, the water was coming from the side of the houses running to the lake, this water was running behind toilets carrying rubbish and all dirty things to the lake. The water went straight to the lake. Some men though it was running they remained at their tent playing cards. Still after the rain people started coming to fetch water.

**2017.11.04 Bugoto Ob LM**

On the 3^rd^ of November I had slept at the health Centre doing observations while they were administering treatment to children. These were kept for a night because the medical team wanted to monitor them as they were to be bled three times. In the morning on 4^th^ as early as 6:00am I had woken up, having slept on a bench with my rain coat as the blanket, when I woke up I just moved out, washed my face using mineral water and went straight to the lake.

By this time the lake is active with many people, as some are leaving others are coming to the lake for different activities especially fishing and fetching water, some stay at the lake shore waiting for those who went for fishing the previous night. At the lake I could see the lights put on the boats lighting. In total I saw 32 boats on the lake, I was told those boats slept over while trapping silverfish and they were to come back in the morning and people at the lake shore were waiting for them, seven people were at the lake fetching water, three boats set off at that time, majority of those who came to fetch water washed their faces first before collecting water, I think they never washed their faces at home since they were going to the lake, three men were standing just at the lake shore doing nothing, I was told those standing were waiting for the fishermen who come back from fishing in the morning. The fourth boat also set off as two boat landed, these boats were joined by those who were standing at the lake to push it to the shoreline. While here I saw men who were coming to the lake with oars and after reaching they pushed their boats into the lake and then set off. I saw more four boats set off as one boat was landing. One woman came at the lake to fetch water; two men were standing in the lake water pulling the net to the lake shore.

At 6:10am I saw 32 people who had come to fetch water and had left, people kept on coming and leaving the lake to fetch water, I saw one woman was organising the nets to put on silver fish and she was seated at the lake waiting for the fisher men. Other three boats landed. By 6:20am the number of those who had come to fetch water had reached 54, I also saw three motorcycles that were brought at the inland area to be washed and they washed them from the lake. Three boats also landed and six people with a lady on went to help in pushing the boat to the lake shore. By 6:30am they were now 86 people who had come to fetch water from the lake, one boat set off as other four boats were landing. By 6:40 the number of those who had come to fetch water was 111. By 7:00am the number of people had reached 172 and by 7:10 the number was 192. These people all entered the lake while fetching water. Two women were drying silver fish, had already spread them on the nets.

**2018.01.30 Bwondha Tw LM**

We left this place and continued following the shore line, I found a bottle of Anti-retroviral therapy at the lake shore, the smell remained bad, the water in this area was black and dirty due to rotten roots of water hythene an open defecation. I found a bush at the lake shore and it was full of faeces, in the bush there was a boy defecating, at the extreme end of this bush there was a man washing who I later found out that he was the father of the two children, there was fresh faeces at the lake shore line and I think it was this child who did open defection.

**2018.01.31 Bwondha Tw LM**

I continued with my walk and found garden irrigated, I guess they got the water from the lake, we proceeded and reached the area they call (Mabanda) this is the place of open defecation and bathing. This place is near a garden and the garden is fenced, I think to fence the garden he feared these people who do open defecation, the place has stones in the lake and a bush at lake shore, it smells bad and everywhere there were faeces both on stones in the lake and the bush at the lake shore, by the time I reached I found man bathing and he had come from the bush around, I think he defecated first, the stones around all had faeces. This very area where they defecate is where they bath from, in case of water waves the open defecation at lake shore can be washed to the lake.

This place was smelling a lot, I could not be here for long, I continued to another place though passing through a bush then to a water melon plantation and finally to another area where people wash from, bath, swim and hook. I still found faeces on my way to this place. When I reached in this place I found over 80 people, majority were washing, children were swimming and bathing, a few hooking and the others were resting and seated just conversing. Some were washing from the lake shore and others were washing directly from the lake. I mostly found women and children this side. One boy was standing in water hooking like 100 meters in the lake. Two men were disentangling nets. Most children were busy hooking while standing in water, others on stones but majority of them were necked while hooking, when I asked why I was told they first swim before hooking and leave clothes at the lake shore. There were a few boats and four men were organizing fishing nets to go for fishing. This area has a bush and stones. The stones and the bush had faeces.

For more data, related to this study please see the supplementary file for another paper [1] from this study published in PLOS Neglected Tropical Diseases.

References:

1. Ssali A, Pickering L, Nalwadda E, Mujumbusi L, Seeley J, & Lamberton P H. (2021). *Schistosomiasis messaging in endemic communities: Lessons and implications for interventions from rural Uganda, a rapid ethnographic assessment study.* *PLoS* Negle Trop Dise, 15(10), e0009893.
