# Supplementary material for: Understanding perceptions of schistosomiasis and its control among highly endemic lakeshore communities in Mayuge, Uganda
Source: PLoS Negl Trop Dis. 2023 Jan 19;17(1):e0010687. doi: 10.1371/journal.pntd.0010687 (PMC9888691; doi:10.1371/journal.pntd.0010687)
Supplement: Attachment — Submitted filename: Response to reviewers comments for Mujumbusi et al.docx [file pntd.0010687.s003.docx]

Dear Reviewers and editor,

Thank you for taking your time to review our manuscript (PNTD-D-22-00953) and for your helpful comments. The comments have guided us to significantly improve the manuscript which we hope you now consider suitable for publication. I confirm that all authors have seen and confirmed they are happy with the updated manuscript.

We look forward to hearing from you,

Kind regards

Lazaaro Mujumbusi (on behalf of all authors)

Please see our responses to the individual comments below in bold.

= L25 “over 240 million people infected” This is not anymore true based on the recent global estimation in 2019. Please revise

**Response: After searching the literature there still seems to be differences in these estimtes. A 2019 Deol paper says 140 million people infected, but a Jan 2022 WHO documents says 236M still need preventative treatment, therefore we have edited the number down but also rephrased the sentence to better reflect this.**

Please revise the abstract by covering the key components of the study:

Key observations in the three studied communities

1. Perceptions about symptoms of schistosomiasis

2. Perceptions about the treatment of schistosomiasis

3. Perceptions about how schistosomiasis is contracted

4. Knowledge and views about schistosomiasis onwards transmission and responsibility of

Transmission

5. Knowledge and views about how people can prevent infection and onward transmission.

**Response: The abstract has been revised to cover these key components in the methods and then addressing them in order in the results section.**

Introduction
= L77 – L79 “In this paper we aim …… treatment practices and how they acted on those choices.” This should move to the end of the introduction

**Response: These lines have been moved to the end of the introduction replacing the original ending to reduce repetition.**

= The introduction could be shorten to 2 and half pages by avoiding speculations on well-known aspects.

**Response: We have cut the introduction back to just over 2.5 pages and agree that it now reads more succinctly and has improved the paper. Thank you.** **We have also cut the methods secton back a bit too.**

Findings

= L303: What is the meaning of “1.”

**Response: This ‘1’ after Figure 3 in the findings refers to the first of the 5 main aims of the paper. We have now made this and the heading underlined to try to make this clearer.**

= shorten the findings section by avoiding multiplying verbatim for the same subject

**Response: We have shortened the results by removing repeated quotes and rephrasing bits to be more succinct.**

You are in the findings section then no discussion. Please, remove the following paragraphs:

= L304 – L306: “In this section we discuss how ……. related to schistosomiasis.”

**Response: This statement has been removed.**

= “In this section, we discuss how …. practices in the use of praziquantel, and traditional medicine.”

= L520 – L521: “In this section, we discuss how ….. prevention impacted control strategies.”

**Response: These statements have been removed.**

Discussion

= L645 “and others supports other qualitative”???

**Response: This has been clarified to now says This supports another qualitative study in lake Victoria communities [17] and a quantitative [36] study also performed in one of these communities.’**

= Discussion is currently six pages and could be shorten avoiding speculation

**Response: we have now cut the discussion down by over a page and agree that the manuscript is greatly improved by this.**

Lines 80-85: references missing

**Response: References for lines 80-85 were in line 86. The incorrect full stops in lines 80-85 made the sentences look as if they are independent of each, and we thank you for highlighting our error which has now been amended.**

Lines 81-82: it should read "actively penetrate the skin and became schistosomule. These enter the circulatory system and migrate to the liver where they develop into adult worms, pair off, and sexually reproduce."
**Response: Thank you for highlighting this error, this section has actually now been cut back when shortening the introduction and is now no longer written like this.**

Line 103: consider putting recent data, as these one are 5 years and older.

**Response: *the available recent publications showing prevalence is for 2019 (Exum, et al.) showing 29.0% was already cited, but labelled as 2017, as that was when they performed their study. We have also added the prevalence of children which was 36.1%.***

Lines 198 and 203-204: the acronym "WaSH" look different, please correct.

**Response: we have checked the manuscript and believe that all WaSH is written as such throughout and as we introduced it.**

Line 276: remove "s" after the word "utensils".

**Response: The "s" has now been removed.**

Lines 289-290: consider moving this statement to Discussion
**Response: This statement has been taken to the discussion.**

Lines 351-352: consider moving this statement to Discussion
**The statement has now been moved to the 6^th^ paragraph of the discussion**

Line 382: correct the spelling for Praziquantel.
**Response: The spelling of praziquantel has been corrected and checked throughout.**

Lines 572-576 and 593-594: consider moving these statements to Discussion.
**Response: Lines 572-576 have been cut (Putting it in the discussion would lengthen the discussion through repetition). Indeed the quote supporting that statement has also been cut. Lines 593-594 have now also been cut.**

Lines 689-740: consider including in the recommendation "improving availability and accessibility of WaSH facilities like safe water, etc", as this is in the results and discussion, not coming out clearly in the recommendations.

**Reponse: This statement has now been included in the discussion to emphasise this point. Thank you.**

Comments
• As well as their knowledge about infection and symptoms, did the communities see SCH as a problem?

**Response: Yes, the community looked at it as a problem and we have now added this in the first paragraph of thefindings**

• Did you record how many of the interviewees had taken PZQ? At last round or ever?

**Response: unfortunately we did not ask them about that.**

• Out of interest, what was the intensity of infection in these areas? Presumably high as well. Were there a lot of people harboring heavy infections?
**Respnse: The intensity of infection in these communities was very high. A paper published by a collegue we work with in this community Arinaitwe et al shows 71% of infection in one of the communities and we have also added this to the methods section under study area in case other readers have the same enquiries.**

Minor / Editorial
• Line 83 – haematobium should be in italics

**Response: This has been amended, thank you.**

• Line 110 – remove extra comma

**Response: The extra coma has been removed, thank you.**

• Line 276 – check typo between ‘utensils’ and ‘other household activities’

**Response: The "s" has now been removed.**

• Figure 4 – typo in title

**The typo in figure 4 legend has been corrected. Thank you.**

**In addition, to the shortening and edits throughout we have also made a minor change to the title adding ‘highly endemic’ for clarity.**
