# Supplementary material for: Understanding perceptions of schistosomiasis and its control among highly endemic lakeshore communities in Mayuge, Uganda
Source: PLoS Negl Trop Dis. 2023 Jan 19;17(1):e0010687. doi: 10.1371/journal.pntd.0010687 (PMC9888691; doi:10.1371/journal.pntd.0010687)
Supplement: Attachment — Submitted filename: Response to reviewers comments for Mujumbusi et al.docx [file pntd.0010687.s004.docx]

Dear Reviewers and editor,

Thank you for taking your time to review our manuscript (PNTD-D-22-00953) and for your helpful comments. The comments have guided us to significantly improve the manuscript which we hope you now consider suitable for publication. I confirm that all authors have confirmed they are happy with the updated manuscript.

We look forward to hearing from you,

Kind regards

Lazaaro Mujumbusi (on behalf of all authors)

Comments.

The correct acronym for Water, Sanitation and Hygiene is WASH not WaSH as used throughout the manuscript. WASH is a UNICEF approved acronym. Please revised the acronym throughout your manuscript including the reference section. Obviously, WaSH is not the same as WASH.

**Response: We have revised and corrected the acronym for Water, Sanitation and Hygiene. It is now written as WASH throughout. Thank you for the vital correction.**
