# Supplementary material for: Understanding perceptions of schistosomiasis and its control among highly endemic lakeshore communities in Mayuge, Uganda
Source: PLoS Negl Trop Dis. 2023 Jan 19;17(1):e0010687. doi: 10.1371/journal.pntd.0010687 (PMC9888691; doi:10.1371/journal.pntd.0010687)
Supplement: Attachment — Submitted filename: Reviewer Comment (2 9 2022).docx [file pntd.0010687.s002.docx]

Author   : Mujumbusi et al.

Title       : Understanding perception of schistosomiasis and its control among lakeshore communities in Mayuge, Uganda.

Journal    : PLoS Neglected Tropical Diseases

Reference: PNTD-D-22-00953

**Abstract**

= L25 “over 240 million people infected” This is not anymore true based on the recent global estimation in 2019. Please revise

Please revise the abstract by covering the key components of the study:

Key observations in the three studied communities

1. Perceptions about symptoms of schistosomiasis

2. Perceptions about the treatment of schistosomiasis

3. Perceptions about how schistosomiasis is contracted

4. Knowledge and views about schistosomiasis onwards transmission and responsibility of

Transmission

5. Knowledge and views about how people can prevent infection and onward transmission.

**Introduction**
= L77 – L79 “In this paper we aim …… treatment practices and how they acted on those choices.” This should move to the end of the introduction

= The introduction could be shorten to 2 and half pages by avoiding speculations on well-known aspects.

**Findings**

= L303: What is the meaning of “1.”

= shorten the findings section by avoiding multiplying verbatim for the same subject

You are in the findings section then no discussion. Please, remove the following paragraphs:

= L304 – L306: “In this section we discuss how ……. related to schistosomiasis.”

= “In this section, we discuss how …. practices in the use of praziquantel, and traditional medicine.”

= L520 – L521: “In this section, we discuss how ….. prevention impacted control strategies.”

**Discussion**

= L645 “and others supports other qualitative”???

= Discussion is currently six pages and could be shorten avoiding speculation
